# Dental Neuralgia

**Published:** 1879-02

**Authors:** G. V. Black


### ARTICLE IV.

### Dental Neuralgia.

BY G. V. BLACK, JACKSONVILLE.

Before proceeding to discuss that form of neuralgia which we designate as Dental Neuralgia, it will be well to briefly consider *neurlgia in general.*

*Neuralgia* is a term used to designate an affection characterized by pain, located in the trunks or branches of the sensory nerves, and unaccompanied by other symptoms. But little is known of its real nature. Post mortem examinations have usually revealed no organic lesion of the nerves involved; the changes, if any, not being of such a nature as to be demonstrable by our present means of examination. There can, however, be but little doubt that in the production of pain there is a molecular excitement, at least in the elements of the nerve itself, that differs essentially from that called into play by the conduction of impressions in the ordinary healthful way. The healthy conduction of impressions, of course, includes pain as well as tactile impressions.

The pain from a wound, or a simple inflammation of a sensitive part—as the tooth-pulp—is conducted to the sensorium in a perfectly healthy way by healthy nerves.

Some forms of neuralgia seem to consist of such a condition of the nerves, that the effort to convey sensation—perform their function—is painful, as the performance of the function of contraction is painful in an inflamed muscle. Other forms of the affection differ from this in that the nerves seem to convey normal sensation properly, and without excitation of pain. It is evident that the nerves are not only the seat of pain, but the seat of disease in many instances.

Neuralgia may be either primary or seconday, but is, perhaps, very generally secondary, and dependent upon some preceding or co-existant diseases or lesion, and, therefore, symptomatic. No division into primary and secondary can be of any value, unless it can be shown that certain symptoms, or classes of symptoms, arise from definite causes, and thus afford a guide to treatment. It is probably best, with our present knowledge of the affection, to always consider it as dependent upon some pre-existant or co-existant malady. This malady or lesion may be of the most obscure character possible, and wholly escape our closest search; indeed, we may say that in a large proportion of cases no cause can be discovered.

The *Pain* in neuralgia is entirely peculiar to this affection, is essentially different in its character and course from that induced by other causes. We may have pain projected from the seat of an injury or inflammation, either in a centric or excentric direction, and yet not constitute neuralgia. We may often find pain projected along the arm, in case of whitlow. The entire arm may become painful; the elbow or the axilla may suffer from projected or sympathetic pain. These sympathetic pains occur from various causes, but are almost always accompanied by intense suffering at the seat of lesion. Certain affections of the teeth, as pericementitis are liable to cause them about the angle of the jaws, throat, &c. The lymphatics are usually involved, and the glands become swollen.

Neuralgic pain is essentially different from these projected pains and seems to have nothing in common with them. The pain is never steady, but presents well marked intermissions or remissions and exacerbations; it is paroxysmal, the paroxysms follow each other in rapid succession, or the intervals may be much extended, even to days, presenting the utmost irregularity. It is usually a sharp, piercing pain, shooting along the course of the nerve, often with lightning-like speed, but sometimes changing its locality slowly, or possibly remaining stationary for a short time in one spot, or skipping about from point to point. We have seen patients endeavoring to point out the immediate seat of pain, place the finger on the chin, under the eye, the angle of the jaw, the ear, the temple, &c., in rapid succession, and declare that the pain ceased in the first locality and appeared in the second, and so on, only one point being painful at the same moment. Again, we have seen the course of the nerves accurately traced in following the pain, as it darted from point to point. Again, the pain may become at once intense along the course, or the portion of a course of a nerve trunk, and remain so for an indefinite time when it will remit or wholly intermit to be renewed after an indefinite time in exactly the same locality. The pain is usually described as sharp, lancinating, piercing, boring, tearing, burning, and the like. Very rarely do we hear patients complain of heavy, dull pain, except in the remission of the paroxysms.

The pain may be seated in the skin, and, therefore, superficial. In this case it is oftenest described as prickling, as though a thousand needles were pricking the flesh. Sometimes this is burning, as though each individual needle was red hot. The slightest causes are often sufficient to induce a paroxysm, as a breath of cold air, the hand passed over the part, or anything that may call upon the affected nerves to convey an impression—exercise their functions—which gives a striking resemblance to the pain induced by the contraction of an inflamed muscle. In other cases the paroxysms come and go without any apparent cause.

There is usually no special attendant constitutional symptom farther than general debility as a result of continued suffering. The disease may, however, and as a fact, is often developed in connection with other diseases, especially such as slowly impair the vital powers, scirrhus, etc.

In many cases, certain painful or tender spots make their appearance along the trunk of the nerve, or at the situation of the ganglia, which are found to be tender on pressure. These tender spots may or may not be the seat of pain. Our personal observation, however, is that they are found to be points from which the pain radiates in the beginning of paroxysms. They are often of diagnostic importance.

*Local Anæsthesia* is frequent result of neuralgia; the nerves seeming to lose, temporarily, the power of conducting impressions. This is perhaps noticed oftenest when the pain affects the surface. In several instances we have seen these anæsthetic spots well marked about the face. We examined a case, recently, in which sensation was entirely lost in the region supplied by the nerves escaping from the mental foramen. Pulps of teeth which cause neuralgia are often found in a state of anæsthesia.

*Etiology.*—The causes of neuralgia may be divided into Predisposing and Exciting. Those who may be predisposed to the affection may have it developed by a very trifling exciting cause, while those who are not so predisposed, such causes may fail entirely to develop the affection.

*Predisposing Causes.*—The Neuropathic predisposition, which has been minutely studied and established in late years, plays a very important part in the causation of many nervous diseases. By this phrase, says Dr. Erb: "is understood a pathological constitution affecting the functional constitution of the nervous system, by virtue of which, those who are thus constituted manifest throughout life the most varied pathological symptoms in regard to sensory, motor and physical processes. No one has yet been able to show in what this peculiar anomaly consists, and while some console themselves with the hypothesis of delicate trophic disturbances, or modifications of molecular arrangement, without thereby getting any nearer the facts, we must rest satisfied that such constitutional Neuropathies really exist, and that they play an important part in the production of neuralgia. They may be congenital, or acquired in consequence of various adverse influences. Many forms of neuralgia owe their origin to transmission from parent to offspring, which seems also true of many other nervous diseases, chorea, hysteria, paralysis, etc."

Certain periods of life also have an influence in predisposing to neuralgia. It does not occur often in childhood, it seems to increase with increasing age. The sexual periods, as puberty, the menopausis, etc., seem also to exert an influence on the nervous system favorable to the development of the affection. Disturbances of nutrition also predispose to it. There is, perhaps, no more fruitful cause than anæmia, under the influence of which many distressing cases are developed.

All influences which tend slowly to depress the vital powers, such as over-work, too little sleep, close application to study, to the needle in sewing women, confinement in illy ventilated rooms, etc., favor the development of the affection.

*Exciting Causes.*—The most prominent exciting causes of neuralgia are wounds, mechanical irritants, punctures, contusions, etc. The severest forms of neuralgia have occurred from these when they have affected only a small branch of a sensory nerve. Irritants applied to a nerve, as a bit of glass, a brier, an exposed tooth-pulp, etc., have been demonstrated to have been the exciting cause of wide-spread neuralgia, not confined to the injured nerve, or its trunk, but radiating to many others besides.

Many cases of neuralgia are also supposed to be due to some changes in the bony orifices through which they pass from their origin to their distribution—to little ossific nodules or exostosis impinging upon the nerve trunks.

Malaria is also a very frequent cause of the affection, giving rise to many very severe forms of the disease. It may act as a predisposing cause in connection with mechanical irritants, or as an exciting cause in connection with other predisposing causes. Very many exciting causes might be noted, but space forbids. Enough has been mentioned to illustrate tendencies from which judgments may be formed in special instances.

[TO BE CONTINUED.]

